# A systematic review of mental disorder, suicide, and deliberate self harm in lesbian, gay and bisexual people

**DOI:** 10.1186/1471-244X-8-70

**Published:** 2008-08-18

**Authors:** Michael King, Joanna Semlyen, Sharon See Tai, Helen Killaspy, David Osborn, Dmitri Popelyuk, Irwin Nazareth

**Affiliations:** 1 Department of Mental Health Sciences, Royal Free and University College Medical School, Hampstead Campus, University College London, London, NW3 2PF, UK; 2 Camden and Islington Mental Health and Social Care Trust, St Pancras Hospital, London, NW1, UK; 3 Department of Primary Care and Population Sciences, Royal Free and University College Medical School, Hampstead Campus, University College London, London, NW3 2PF, UK; 4 General Practice Research Framework, Medical Research Council, 158-60 North Gower Street, London, NW1 2ND, UK

## Abstract

**Background:**

Lesbian, gay and bisexual (LGB) people may be at higher risk of mental disorders than heterosexual people.

**Method:**

We conducted a systematic review and meta-analysis of the prevalence of mental disorder, substance misuse, suicide, suicidal ideation and deliberate self harm in LGB people. We searched Medline, Embase, PsycInfo, Cinahl, the Cochrane Library Database, the Web of Knowledge, the Applied Social Sciences Index and Abstracts, the International Bibliography of the Social Sciences, Sociological Abstracts, the Campbell Collaboration and grey literature databases for articles published January 1966 to April 2005. We also used Google and Google Scholar and contacted authors where necessary. We searched all terms related to homosexual, lesbian and bisexual people and all terms related to mental disorders, suicide, and deliberate self harm. We included papers on population based studies which contained concurrent heterosexual comparison groups and valid definition of sexual orientation and mental health outcomes.

**Results:**

Of 13706 papers identified, 476 were initially selected and 28 (25 studies) met inclusion criteria. Only one study met all our four quality criteria and seven met three of these criteria. Data was extracted on 214,344 heterosexual and 11,971 non heterosexual people. Meta-analyses revealed a two fold excess in suicide attempts in lesbian, gay and bisexual people [pooled risk ratio for lifetime risk 2.47 (CI 1.87, 3.28)]. The risk for depression and anxiety disorders (over a period of 12 months or a lifetime) on meta-analyses were at least 1.5 times higher in lesbian, gay and bisexual people (RR range 1.54–2.58) and alcohol and other substance dependence over 12 months was also 1.5 times higher (RR range 1.51–4.00). Results were similar in both sexes but meta analyses revealed that lesbian and bisexual women were particularly at risk of substance dependence (alcohol 12 months: RR 4.00, CI 2.85, 5.61; drug dependence: RR 3.50, CI 1.87, 6.53; any substance use disorder RR 3.42, CI 1.97–5.92), while lifetime prevalence of suicide attempt was especially high in gay and bisexual men (RR 4.28, CI 2.32, 7.88).

**Conclusion:**

LGB people are at higher risk of mental disorder, suicidal ideation, substance misuse, and deliberate self harm than heterosexual people.

## Background

Lesbian, gay and bisexual (LGB) people appear to be at greater risk than heterosexual people of mental disorders and suicidal behaviour [1,2]. LGB people are subject to institutionalised prejudice, social stress, social exclusion (even within families) and anti-homosexual hatred and violence and often internalise a sense of shame about their sexuality [1,2]. Lifestyle factors such as alcohol and drugs misuse also increase the risk of morbidity [1] as well as suicide attempts [3]. Deliberate self harm (DSH) is intentional self poisoning or injury, irrespective of the apparent purpose of the act. DSH is one of the leading causes of acute medical admissions in the UK. Incidence rose steadily from the mid 1980s to the late 1990s with a peak incidence rate of 400 per 100,000 per annum [4], one of the highest in Europe. However there is evidence of a steady drop in suicide in England (and other developed countries) since 2000 [5]. The evidence on mental health of LGB people is inconclusive partly because of the difficulty of defining or recruiting samples that are representative of all non-heterosexual people. Specific methodological obstacles include variation in the definition of sexual orientation, DSH and mental illness; difficulty in achieving random samples; reliance on participants' recall; unwillingness of people to be open about their sexual orientation; lack of information on sexuality in suicide victims who are part of psychological post mortem studies; the complexity of choosing appropriate comparison groups and poor or absent adjustment for confounding influences such as substance use and personality factors.

There is an urgent need to quantify the risk for mental disorder, DSH and suicide in LGB people, to understand the precipitants and to examine the efficacy of prevention efforts. There is also a need to make a judgement about the quality of the evidence available. We undertook a systematic review of the world literature on risk of mental disorder, substance misuse, DSH, suicidal ideation and suicide in LGB people. These parameters are the main ones reported in the literature and provide a comprehensive picture of mental health and well being.

### Hypothesis

Gay, lesbian and bisexual people have higher risks than heterosexual people of mental disorder, substance misuse and dependence, suicide, suicidal ideation and DSH.

### Objective

To undertake a systematic review of the international research literature to establish whether LGB people are at higher risk of mental disorder, substance misuse, suicide, suicidal ideation and DSH than heterosexual people and to quantify this risk.

## Method

We searched for studies of mental disorder, drug and alcohol misuse and dependence, DSH, suicidal ideation and/or suicide in general (community) or selected (e.g. student) populations in which sexual orientation was reported. We use the following abbreviations: GB (gay and bisexual men); LB (lesbians and bisexual women) and LGB (lesbians, gay men and bisexual men and women).

### Data sources

We searched Medline, Embase, PsycInfo, Cinahl, the Cochrane Library Database, the Web of Knowledge, the Applied Social Sciences Index and Abstracts, the International Bibliography of the Social Sciences, Sociological Abstracts, the Campbell Collaboration and grey literature databases for articles published between January 1966 and April 2005. We searched all terms related to homosexual, lesbian and bisexual people and all terms related to mental disorders, suicide, and deliberate self harm. No language limits were imposed. A full internet search was also carried out using Google and Google Scholar and authors were contacted where necessary. We also searched the reference lists of relevant papers.

### Study selection

#### Eligibility

We included papers that provided valid definition of sexual orientation and mental health outcomes. Random sampling is hampered by participants' reluctance to disclose their sexual orientation and the small numbers of LGB people recruited. Thus other methods such as snowball sampling (initial LGB participants recruit other LGB people in successive waves) were regarded as acceptable if the study met other inclusion criteria. We included studies in which people defined themselves as: gay, lesbian, homosexual, bisexual and/or in which they reported levels of same sex attraction or behaviour. We excluded studies based in clinical or psychological services. We only included studies in which there was a concurrent heterosexual comparison group within either a cohort, case-control or cross sectional study. Outcomes were defined as: a) a psychiatric disorder according to the International Classification of Diseases or the American Psychiatric Association's Diagnostic and Statistical Manual (including substance misuse disorders); b) scores or a recognised threshold for psychiatric morbidity on standardised scales (including alcohol or drug dependence); c) alcohol misuse: consumption above UK Government recommended maximum weekly limits (21 units men, 14 units women); d) suicide (the intentional taking one's own life) e) suicidal ideation (i.e. thoughts of taking one's life without acting on them); f) DSH: intentional self poisoning or injury irrespective of the apparent purpose of the act [5]. These outcomes were extracted for both the LGB and heterosexual comparison groups as cumulative incidence rates in prospective cohort studies or period prevalence rates in cross sectional studies.

#### Screening process and assessment of eligibility

The titles and abstracts of citations were screened by JS and DP and those not meeting eligibility criteria, unpublished dissertation theses, case reports, letters, commentaries, or review papers were excluded. Decisions on papers included in the final review were made by pairs of authors and disagreements discussed at steering group meetings involving all authors.

### Data extraction

At least two of the authors extracted data from each paper on study setting, study design, population and sampling details, attrition and response rate. We recorded the definition of LGB sexual orientation (same sex attraction; same sex behaviour; self identification as lesbian gay or bisexual; a score above zero on the Kinsey scale [6]) and outcome (mental disorder, substance misuse, DSH, suicidal ideation and suicide). Where appropriate we extracted prevalence estimates and/or odds ratios; for continuous data we extracted means and standard deviations. In instances of disagreement, each case was discussed by all authors.

### Quality of studies reviewed

We used the Cochrane Handbook's general guidance on non-experimental studies to inform our choice of quality indicators (2 indicating higher quality than 1). We examined for: *sampling*: non random = 1, random = 2; *representativeness*: response rates: <60% = 1, 60% or more = 2; *population definition*: selected sample (e.g. school students) = 1; general population = 2 and *sample size*: <100 LB or GB people = 1, >100 LB or GB people = 2.

### Data synthesis

Studies were grouped according to lifetime or 12 month prevalence and where possible we analysed outcomes for lesbians, gay men and bisexual people separately and collectively. We calculated risk ratios and attributable risks (differences between rates in LGB and non LGB people) from extracted prevalence data. We examined suicide attempts when reported instead of or in addition to DSH. For continuous outcomes we calculated the effect size as standardised mean difference in scores between LGB people and controls.

#### Meta-analytic approach

We adopted standard methods for conducting meta-analyses where there were two or more studies with useable outcome data. We used a random effects model which used inverse variance methods to calculate the pooled effect estimate in which the weight given to each study is the inverse of the variance of the study estimate together with the common heterogeneity variance. We quantified the effect of heterogeneity [7] by using *I*^2 ^which describes the percentage of total variation across studies that can be attributed to heterogeneity rather than chance [8].

## Results

**Figure 1:**
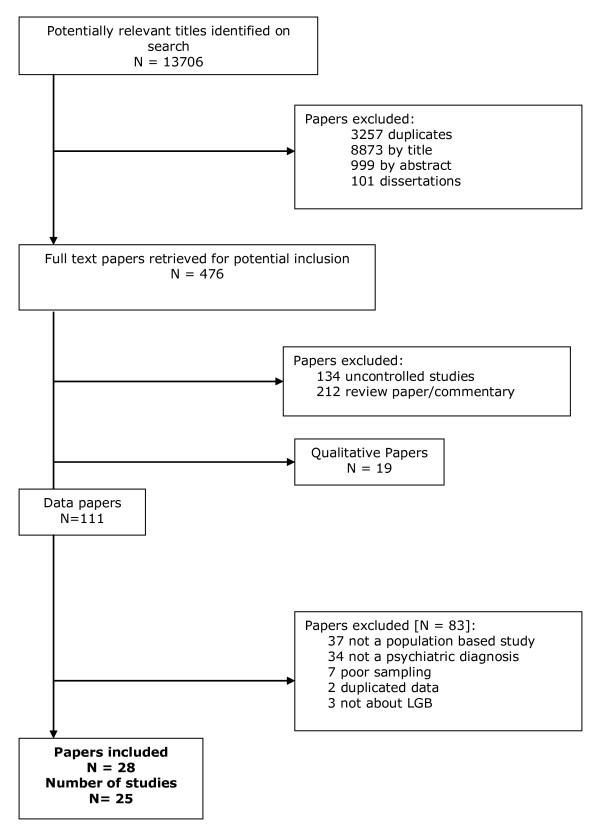
Study inclusion process.

From 13706 citations identified, 476 papers were retrieved of which 429 were excluded (figure 1). Eighty-three of those excluded were controlled studies [36-120], 122–123]; two [39,40] were excluded because the data were repeated elsewhere [29]; seven did not meet sampling criteria [36-38,75,76,118,119]; 34 did not report suicide, DSH or diagnostic outcomes [41-74]; 37 involved unrepresentative populations [77-113] and three on closer inspection did not concern LGB people [114-116]. There were insufficient data in three studies on completed suicide to include it as an outcome in the review. One that involved suicide in a cohort of bisexual and gay men was excluded because it was uncontrolled [117]; one study comparing clinical characteristics of a subpopulation of gay and non-gay male suicides was excluded because of sampling concerns [118] and a psychological autopsy study carried out in 1995 [119] was excluded as it contained only three gay male suicides.

### Study characteristics

Twenty-eight papers [1,9-35] reporting on 25 studies [1,9-12,14,15,17-31,33-35] met our inclusion criteria (Additional file 1); six papers [12,13,15,16,31,32] reported data on three studies. Five studies could not be included in a meta-analysis because the data were not extractable or in a format that allowed comparison [10,11,22,27,34]. Three of the four longitudinal cohorts [11,18,33] presented nested cross-sectional data on sexual orientation and mental health at one time point. One cohort study, however, conducted a longitudinal analysis of cumulative incidence of suicidal attempts but did not provide extractable data [34]. No case-control studies were identified. The studies were conducted in seven countries in North America, Europe and Australasia, with most based in the USA (17/25, 67%). The papers were published between 1997 and 2004, with two thirds published between 2000 and 2003. Participation rates ranged from 25% [23] to 95% [28].

### Population

The papers contained data on 214,344 heterosexual and 11,971 non heterosexual people aged 12 and over. Four studies involved people aged under 18 [10,17,29,30] and 18 involved people under 25 years. Four studies included only women [11,20,24,26], three only men [9,14,21] and 18 both sexes. Eight studies [10,17,21,25,26,28-30,34] concerned high school and college students. Of the 21 cross sectional studies, nine used random sampling [9,15,19,20,22,25,26,31,35]; two multi-stage sampling [12,14]; two snowball sampling [1,24]; one systematic sampling (i.e. 26 years follow up data on a birth cohort) [23]; and seven did not specify their sampling method [10,17,21,27-30].

### Definition of sexuality

Sexuality was defined in a number of ways even within the same study: four studies used same sex attraction [24,30,33,34]; 13 used same sex behaviour [9,10,12,14,17-19,21,24,29-31,34,35]; 15 used participant self identification [1,9-11,15,18,20,22,23,25-29]; and three used a score above zero on the Kinsey scale [1,28,34] (see Additional file 1). Nine studies used two definitions of sexual orientation [1,9,10,18,24,28-30,35] and one used three definitions [34]. Self-identified sexuality was based on the categories heterosexual, homosexual or bisexual [9,15,18,20,22,23,28] or included the choices gay or lesbian [1,10,11,25-27,29]. Eighteen studies used a specific time frame to assess sexuality. Lifetime same sex attraction was assessed in two studies [30,33]; current same sex attraction assessed in four [24,33-35] and in one study both were assessed [33]. Same sex behaviour was assessed as occurring 'in the last year' in two studies [12,24], 'in the last five years' in one study [19] or 'ever' in nine studies [9,10,14,17,18,21,29,30,34].

### Outcomes of interest

**Figure 2:**
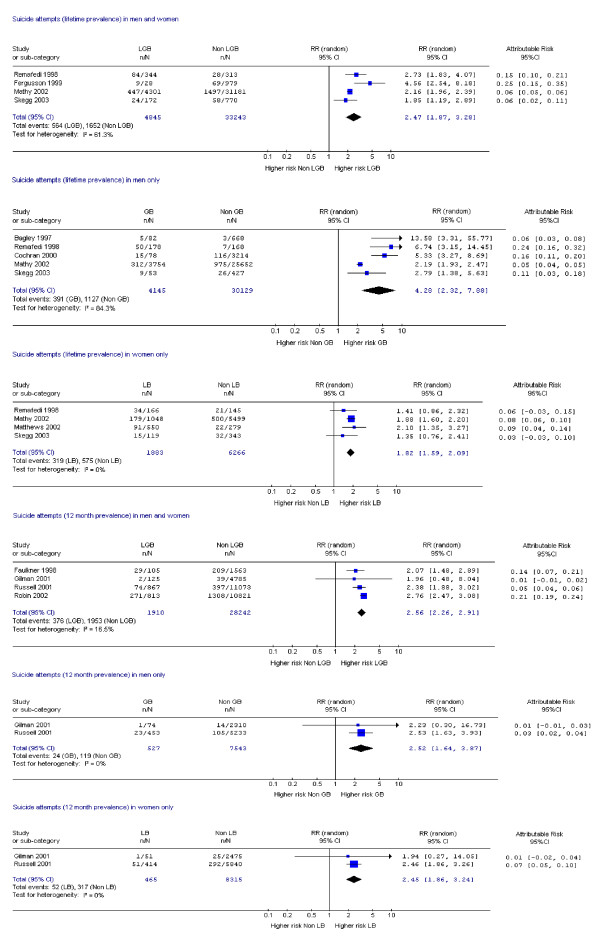
Forest plots for lifetime and 12 month prevalence of suicide attempts.

**Figure 3:**
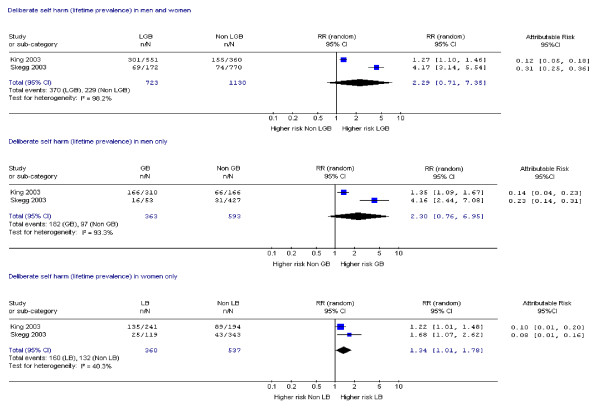
Forest plots for lifetime prevalence of deliberate self harm.

**Figure 4:**
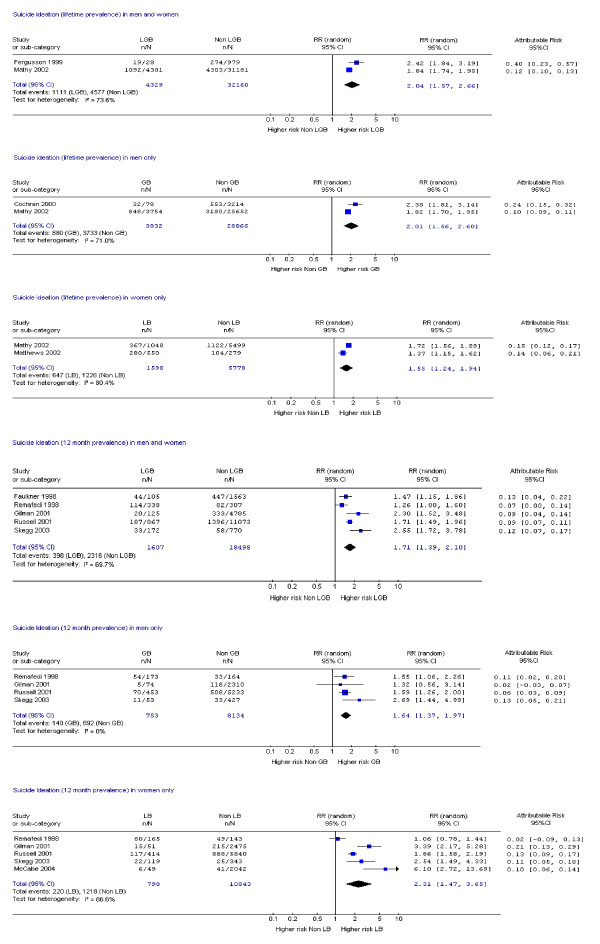
Forest plots for lifetime and 12 month prevalence of suicide ideation.

**Figure 5:**
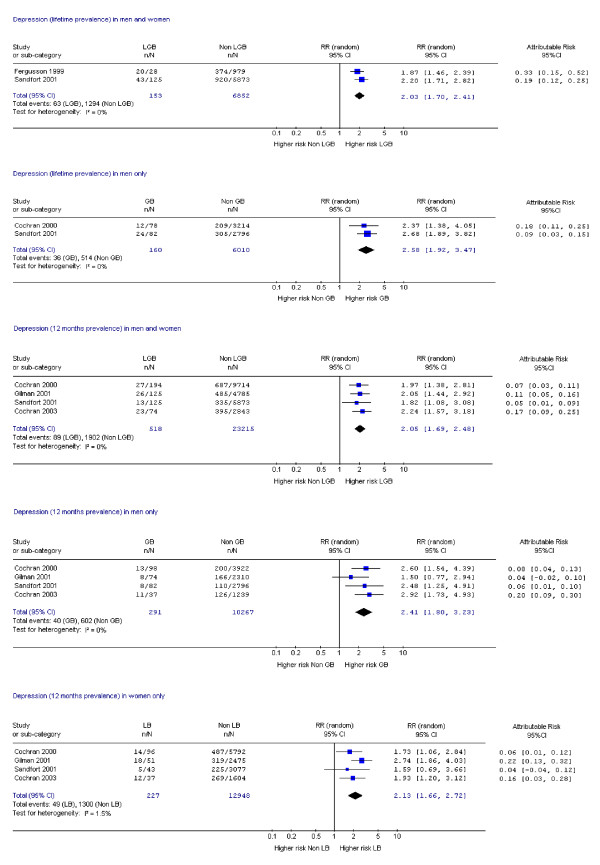
Forest plots for lifetime and 12 month prevalence of depression.

**Figure 6:**
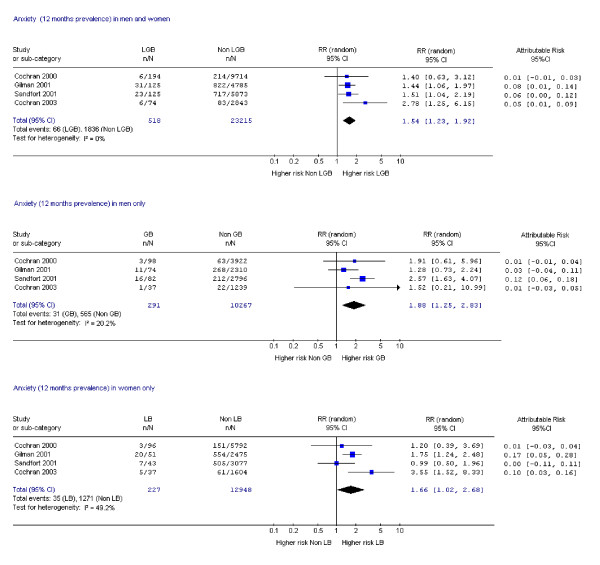
Forest plots for 12 month prevalence of anxiety.

**Figure 7:**
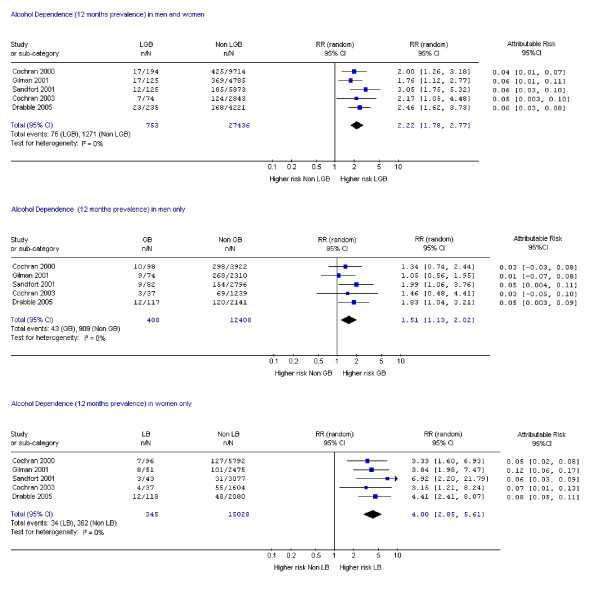
Forest plots for 12 month prevalence of alcohol dependence.

**Figure 8:**
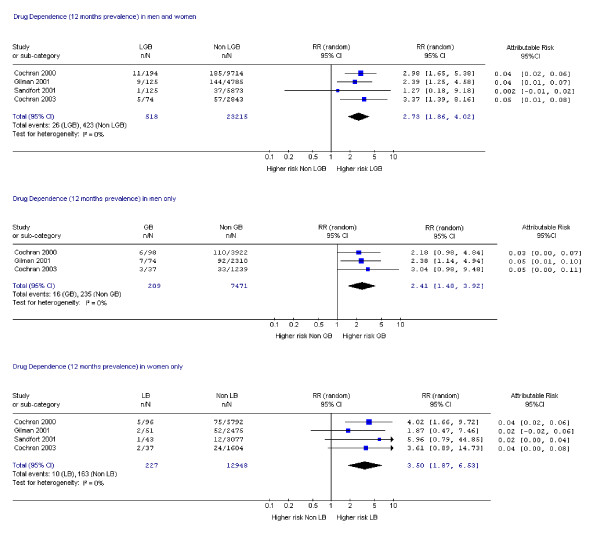
Forest plots for 12 month prevalence of drug dependence.

**Figure 9:**
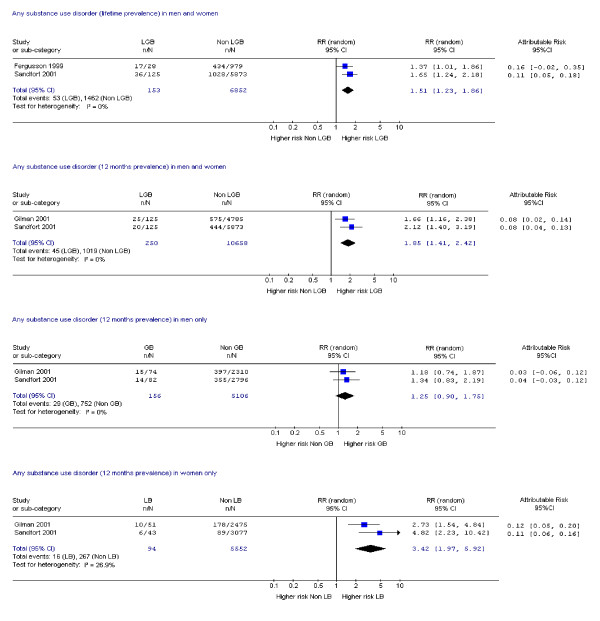
Forest plots for lifetime and 12 month prevalence of any substance use disorder.

Fifteen studies assessed suicide attempts or DSH [1,9,10,14,17-19,21,23,24,28-30,33,34] and 12 assessed suicidal ideation [14,17-19,21-24,26,28,30,33]. Data on mental disorder were assessed in 10 studies [1,9,11,12,14,15,18,19,22,31], substance dependence in six studies [12,15,18,19,31,35] and substance misuse in nine studies [1,19,20,22,25-27,31,35]. Eighteen studies assessed more than one of these outcomes [1,9,12,14,15,17-19,21-24,26,28,30,31,33,35] and one study assessed all [19]. Risk ratios and attributable risks were calculated for all outcomes of interest (figures 2, 3, 4, 5, 6, 7, 8, 9).

### Quality of cross sectional studies

Nine studies were based on random populations but only seven of these were sampled from the community rather than from specific groups (e.g. schools). Only four of these reported responses of at least 60% and of these only one [19] sampled 100 or more LGB people (table 1).

**Table 1:** Classification of quality indicators of studies included in the review

	Sampling	Participation rate	Population	Sample size
	1 = Non-random	1<60%	1 = Selected	1<100
	2 = Random	2 ≥ 60%	2 = General	2 ≥ 100
Bagley 1997 [9]	2	2	2	1
Bontempo & D'Augelli 2002 [10]	Not known	2	1	2
Case et al. 2004 [11]*	1	2	1	2
Cochran & Mays (2000a) & Mays, Ross (2004) [12,13]	1	2	2	1
Cochran & Mays (2000b) [14]	1	2	2	1
Cochran et al. 2003 & Mays & Cochran (2001) [15,16]	2	2	2	1
Faulkner et al. (1998) [17]	Not known	1	1	2
Fergusson et al 1999 [18]*	1	2	1	1
Gilman et al. 2001 [19]	2	2	2	2
Gruskin et al. 2001 [20]	2	1	2	2
Herrell et al. 1999 [21]	Not known	2	1	2
Jorm et al. 2002 [22]	2	1	2	2
King et al. 2003 [1]	1	NA	1	2
Mathy 2002 [23]	1	1	1	2
Mathews et al. 2002 [24]	1	1	1	2
McCabe et al. 2003 [25]	2	1	1	2
Mc Cabe et al. 2004 [26]	2	1	1	1
Nawyn et al. 2000 [27]	Not known	1	1	1
Remafedi et al. 1998 [28]	Not known	2	1	2
Robin et al. 2002 [29]	Not known	2	1	2
Russell & Joyner 2001 [30]	Not known	Not known	1	2
Sandfort et al. 2001 & Sandfort, de Graf, Bijl (2003) [31,32]	2	2	2	1
Skegg et al. 2003 [33]*	1	2	1	1
Wichstrom & Hegna 2003 [34]*	1	2	1	2
Drabble et al. 2005 [35]	2	Not known	2	2

Key: * Longitudinal studies; 2 suggests higher quality and than 1

### Data syntheses

#### Suicide attempts and DSH

Only one cohort study [34] reported cumulative incidence of suicide attempts over two years in 2924 Norwegian school youths. They reported an odds ratio of 4.69 (95% CI 2.29, 10.62) for LB girls after adjustment but no significant differential for BG boys.

Meta-analyses of cross-sectional studies of lifetime suicide attempts demonstrated increased risk in all groups when compared to heterosexuals but there was substantial heterogeneity when these data were combined for both sexes and for men only (Figure 2). Attributable risk ranged from 0.03 to 0.25 and was higher in men than women. Studies in this analysis were limited by small samples [9,14,18,33] or selection bias [18,23,28,33] (Table 1). One small study that met all but one quality criteria showed a high risk of suicide attempts in men (Figure 2) [9]. Meta-analysis in women demonstrated 1.82 times increased risk of lifetime suicide attempts in lesbians and bisexuals compared to controls and showed little heterogeneity (Figure 2). However, all the studies failed to meet several of our quality indicators.

Risk ratios for 12 month prevalence of suicide attempts ranged from 1.96 to 2.76 (men 2.23 to 2.53; women 1.94 to 2.46), while attributable risk ranged from 0.01 to 0.14 (men 0.01 to 0.03; women 0.01 to 0.07). The pooled estimate for men and women was 2.56 (Figure 2) with similar values for LB and GB people and all showed little or no heterogeneity. The highest quality study [19], however, showed a non significant risk ratio for all groups.

Only two studies reported lifetime prevalence of DSH [1,33] (Figure 3) and meta-analyses of these data produced equivocal results. One further study that met all but one of our quality criteria reported elevated risk of lifetime prevalence of DSH and/or suicide attempts [9] in gay rather than bisexual men (RR: Gay = 3.61, CI 1.86, 7.01; Bisexual men = 1.95, CI 0.73, 5.19).

#### Suicidal ideation

Meta-analyses of lifetime prevalence of suicidal ideation revealed risk ratios of 2.04 for both sexes (range: both sexes 1.72 to 2.42; men 2.0 to 4.10; women 1.75 to 2.10) with considerable heterogeneity. Attributable risk ranged from 0.10 to 0.40 (Figure 4). All studies included in this analysis were limited by selection bias [23,24] and small samples [12,17].

The combined meta-analysis of 12 month prevalence of suicidal ideation contained some heterogeneity in both sexes and in women, but none in men. The risk ratio in both sexes was 1.71 (men 1.64; women 2.31) while attributable risk ranged from 0.02 to 0.21 (men 0.02 to 0.13; women 0.02 to 0.21). One study that met all four quality criteria [19] demonstrated over three times the risk in women but not in men. The other studies were limited by selection of very young populations [17,28,30,33,26] or low participation rates [26].

In summary, there were elevated risks for suicide attempts and ideation in LGB people but quality of studies was limited. Data from higher quality studies showed higher cumulative incidence of suicide in LB school girls, increased lifetime risk of suicide attempts in GB men and increased 12 months risk of suicidal ideation in LB women.

#### Mental disorders – depression

Three studies reported lifetime prevalence of depression [14,18,31]. Increased risk of lifetime depression was observed in both sexes and men with little heterogeneity in the analyses (Figure 5). One of the two studies that met all but one quality criteria demonstrated a risk ratio of 2.2 in both sexes; 2.68 in men (Figure 5); and 2.21 (CI 1.57, 3.12) in women [31].

The risk of 12 months prevalence of depression in LGB people on meta-analysis was at least twice that of heterosexual controls with little heterogeneity (Figure 5). All studies in this analysis were of good quality based on general population samples with high participation rates. Risk ratios ranged from 1.57 to 3.74 (men 1.57 to 3.74; women 1.67 to 3.69) and attributable risk from two studies ranged from 0.04 to 0.20 (men 0.04 to 0.20; women 0.04 to 0.22). The only study that met the highest standard on the four quality criteria demonstrated significantly higher risk ratios and attributable risk for women but not men [19]. Lastly, a study of 45 gay and 37 bisexual men that recorded depression on a standardised scale and met all but one of our quality criteria showed a small but positive effect size indicating more depression in gay or bisexual men (standardised mean difference in depression score 0.16) [9].

#### Mental disorders – anxiety

Two studies reported lifetime prevalence of any anxiety disorder and both met all but one of the quality criteria [18,31]. Although their data could not be combined in a meta-analysis, increased risk was reported in both sexes (RR 2.28 CI 1.25, 4.21) [18] and in men (RR 2.40, CI 1.72, 3.35) [31], but not in women (RR 1.02, CI 0.61, 1.70) [31]. The meta-analyses of data on 12 month prevalence of any anxiety disorder (Figure 6) resulted in a pooled RR of 1.54 for both sexes and 1.88 in men with little heterogeneity. Attributable risk ranged from 0.00 to 0.17 (men 0.01 to 0.12; women 0.00 to 0.17). The result in women was less convincing because of heterogeneity. The only study of the four in this analysis that met the highest of all four of our quality criteria demonstrated an elevated risk of 1.75 in women [19]. All the studies were based on general population samples and were of reasonable quality.

In summary, on the basis of studies of relatively good quality, there was an elevated risk of lifetime and 12 month prevalence of depression and anxiety disorders in all LGB groups compared to heterosexual controls.

#### Alcohol misuse

Data from a single study that met all but one of our highest quality criteria showed increased risk of lifetime prevalence of alcohol dependence in both sexes (RR 2.59 CI 1.62, 4.15) and women (RR 6.51, CI 2.74, 15.44) but not in men (RR 1.60, CI 0.91, 2.80) [31]. All the studies in this analysis met at least three of our four quality criteria. Risk ratios for alcohol dependence in the previous 12 months in both sexes ranged from 1.76 to 3.05 and were higher in women (Figure 8). Attributable risk for alcohol dependence over 12 months was higher in women (Figure 8). Two studies presented data in accordance with our definition of alcohol misuse within the previous 12 months. McCabe et al (2003) [25] reported little difference between LGB people and controls, but Gruskin et al (2001) [20] reported higher risk of alcohol misuse (RR 3.52, CI 1.97, 6.26) in LB than heterosexual women, with an attributable risk of 7%.

#### Drug misuse or any substance misuse disorder

One study reported higher risks of lifetime prevalence of drug dependence in both sexes (RR 4.32, CI 2.14, 8.72), men (RR 2.71, CI 1.01, 7.37) and in women (RR 7.74, CI 2.88, 20.75) [31]. Meta-analyses of data on drug dependence over the previous 12 months showed 2.73 times greater risk in both sexes, 3.5 times greater in women and 2.41 times greater in men than controls (Figure 9). Attributable risk for drug dependence in the previous 12 months ranged from 0.002 to 0.05 in both sexes, in men 0.03 to 0.05 and women 0.02 to 0.04 (Figure 9).

One good quality study [31] of lifetime prevalence of any substance use disorder showed elevated risk in women (RR 3.61 CI 2.13, 6.11, attributable risk 0.11 to 0.26) but not men (RR 1.05, CI 0.76, 1.47; attributable risk -0.08 to 0.11). Similar findings arose in the meta-analyses of data from two good quality studies on 12 months prevalence of any substance use disorder (figure 9).

In summary, there was an increased lifetime and 12 month risk of alcohol and drug dependency in all groups compared with heterosexuals with markedly higher risk in lesbian and bisexual women.

## Discussion

LGB people are at higher risk of suicidal behaviour, mental disorder and substance misuse and dependence than heterosexual people. The results of the meta-analyses demonstrate a two fold excess in risk of suicide attempts in the preceding year in men and women, and a four fold excess in risk in gay and bisexual men over a lifetime. Similarly, depression, anxiety, alcohol and substance misuse were at least 1.5 times more common in LGB people. Findings were similar in men and women but LB women were at particular risk of substance dependence, while lifetime risk of suicide attempts was especially high in GB men.

### Strengths and limitations of the review

We found 25 studies that met our inclusion criteria for epidemiological rather than clinical studies. Our search terms included all possible subcategories of mental disorder and substance dependence. We identified a wide range of study methods but excluded designs that provided biased or erroneous estimates. We included studies with consistent definitions of sexual orientation and with contemporaneous comparison groups. However, the lower than expected prevalence of LGB people in several of the population surveys [27,31,32] indicates that many studies were unable to recruit a representative sample. Thus, it is likely that a proportion of LGB people are reluctant to participate in research for all sorts of reasons, but most likely for fear of disclosure. Until it becomes less risky to identify oneself as LGB for the purposes of research we shall know little about this hidden population or how it influences the conclusions we can make here. All studies used well-described and potentially replicable mental health outcomes. However, only one study met all four of our quality criteria, while seven met all but one of our quality markers. The number of studies in each meta-analysis was relatively small and thus we were unable to interpret funnel plots to investigate sources of bias or run a meta-regression analysis to account for the variable quality of the studies identified in this review.

Given the range of study design and definitions of exposure and outcome, we encountered significant heterogeneity in our meta-analyses. However, these estimates did not deviate markedly from data reported in the better quality studies. Although, in some studies reported data were weighted or shown as percentages, our calculated risk ratios were similar to unadjusted ratios reported in these papers making it unlikely that we have extrapolated beyond the studies' findings. The distinction between suicide attempt and DSH was often unclear. We followed authors' definitions of the acts and did not judge the life threatening nature of the behaviour. Finally, uncertainties inherent in defining and recruiting a representative sample of LGB people cannot be overcome in a systematic review. For example, participants may be asked about their sexuality in ways that are unfamiliar to them or it may be assumed that sexual orientation is a fixed life-time characteristic. Despite these reservations about our review, the consistent direction of our findings suggests that mental health is poorer in LGB people.

### Selection of studies

We had to exclude otherwise well conducted research that was based in specialised populations or in health services or that selected LGB people in a particular way. We wish to highlight three studies that we eventually excluded on grounds of selection of the LGB population [36-38]; but whose results were broadly in the direction of our findings. Russell & Keel (2002) [36] reported data on depression using the Beck Depression Inventory; van Heeringen & Vincke (2000) [38] reported data on suicide attempts and ideation and Savin-Williams (2001) [37] reported data on suicide attempts.

### Explanations for our findings

Our study aimed to determine whether there was unequivocal evidence for a preponderance of mental health problems in LGB people relative to heterosexuals. Thus, circumspection is required when discussing possible mechanisms which generate them [120]. Although our evidence does not specify the nature of such mechanisms, there is no evidence to suggest that homosexuality is itself a disorder that is thereby subject to a higher co-morbidity than is found in heterosexuals [120]. This review was strictly limited to documenting whether or not there was an excess of mental health problems in LGB people. It will take other, prospective research to investigate the components of this vulnerability. Unfortunately prospective studies were unusual among the 25 reviewed here and thus we cannot say much with certainty about the risk factors for mental disorder in LGB people. Nevertheless, it is likely that the social hostility, stigma and discrimination that most LGB people experience is at least part of the reason for the higher rates of psychological morbidity observed. This may be aggravated by easy access to alcohol and drugs in gay venues that LGB people frequent both to find the company of others who will accept them less critically and to meet potential partners. However, why LB women are at greater risk of substance misuse than GB men is not clear as most LGB commercial venues provide alcohol.

### Implications of our findings

It is of considerable concern that sexual minorities such as LGB people suffer so many disadvantages in terms of mental health. Our findings need consideration in planning public health and clinical services, as well as in terms of international human rights. Although we cannot report on whether or not LGB people are at greater risk than heterosexuals for completed suicide, the elevated risks for all forms of mental disorder, DSH and substance misuse would suggest very strongly that this is the case. Thus, national suicide strategies need to include LGB people as a high risk group now rather than await more evidence on suicide. The hidden nature of sexual orientation makes it very unlikely that we shall be able to show definitely in post-mortem psychological studies that LGB are over-represented among suicide victims.

## Conclusion and further research

Besides more qualitative and case-control research, we need prospective studies as these are most likely to reveal the mechanisms involved. Although, in this review we identified four cohorts [15,18,33,34] only one collected prospective data on suicidal risk in lesbian, gay and bisexual people [34]. Prospective studies, however, are difficult to undertake as many people cannot or will not identify themselves as LGB until late adolescence or even young adulthood when the emotional damage may already have occurred. Nevertheless, a cohort of young LGB people who are followed through as they complete education and career training and start relationships and families, would begin to address this difficult issue. We also need to address the complexities of defining sexual orientation. Most modern conceptions of sexual orientation consider personal identification, sexual behaviour and sexual fantasy [121]. Thus, we chose these parameters as the most pragmatic and commonly used definitions for this review. However, we need more detailed study of the development of sexuality across the spectrum of partner preference, its stability over time and its relationship to other preferences and behaviour.

## Competing interests

The authors declare that they have no competing interests

## Authors' contributions

MK, HK, DO, IN and SST obtained funding for the study. JS and DP conducted the literature search, obtained papers and extracted data. JS, MK, HK, DO, IN and DP scanned abstracts and read papers. SST conducted the meta-analysis with input from IN and MK. MK drafted the paper and all authors contributed to the final version. All authors read and approved the final version.

## Pre-publication history

The pre-publication history for this paper can be accessed here:



## Supplementary Material

Additional File 1 Table 1: review studies. Click here for file
