# Supplementary material for: A systematic review of mental disorder, suicide, and deliberate self harm in lesbian, gay and bisexual people
Source: BMC Psychiatry. 2008 Aug 18;8:70. doi: 10.1186/1471-244X-8-70 (PMC2533652; doi:10.1186/1471-244X-8-70)
Supplement: Additional File 1 — Table 1: review studies. [file 1471-244X-8-70-S1.doc]

**Table 1: Review studies**

| Study Description  Author/Year/  Country | Nature of population | Sampling/recruitment technique  and response rate | Numbers of participants | | | Definition of sexual orientation[[1]](#footnote-2) | Study outcomes of interest[[2]](#footnote-3) |
| --- | --- | --- | --- | --- | --- | --- | --- |
| Bagley & Tremblay (1997) [9]  Canada | Men aged 18-27 years. | Stratified random representative population telephone sample. Response rate 73% | 45 Gay Men  37 Bisexual Men | | 668[[3]](#footnote-4) Non GB Men | 2, 3 | **Suicide attempt[[4]](#footnote-5) [lifetime]**  Depression  (CES-D[[5]](#footnote-6)) |
| Bontempo & D'Augelli (2002) [10]  USA | Male and female students aged 14-17 years. | Representative sample of high school students drawn from the 1995 Risk Behaviour Survey conducted in public high schools in two states.  Response rate 60% | 119 LB Women  196 GB Men | | 4457 Non LB Women  4416 Non GB Men | 2, 3 | Suicide attempt  [<12 months] |
| Case, Bryn, Austin Hunter, Manson, Malspeis, Willett et al (2004) [11]  USA | Female registered nurses aged 25-43 years. | Nurses’ Health Study II cohort (began 1989). Registered female nurses that answered sexual orientation question in 1995. Response rate 79% | 694 Lesbians  317 Bisexual Women | | 89912 Non LB Women | 3 | Depression (SF-36[[6]](#footnote-7)+)  [<12 months] |
| Cochran & Mays (2000a) [12], Mays, Ross (2004) [13] USA | Community sample of men and women aged 18+ years | Complex, stratified, multi stage cluster sampling of general US population >18 years old with a sexual partner in the previous year.  Response rate 80%[[7]](#footnote-8)∇ | 96 LB Women  98 GB Men | 5792 Non LB Women  3922 Non GB Men | | 2 | **DSM-IV Major Depression [<12 months and lifetime]**  **DSM-IV Generalised Anxiety Disorder**  **[<12 months]**  **DSM-IV Alcohol Dependence [<12 months]**  **DSM-IV**  **Drug Dependence [<12 months]** |

| Study Description  Author/Year/  Country | Nature of population | | | Sampling/recruitment technique  and response rate | | | Numbers of participants | | | | | | | Definition of sexual orientation | | | Study outcomes of interest |
| --- | --- | --- | --- | --- | --- | --- | --- | --- | --- | --- | --- | --- | --- | --- | --- | --- | --- |
| Cochran & Mays (2000b) [14]  USA | Men aged 17-39 years with information on psychiatric health and partner gender. | | | Complex, multi-stage sampling of civilian non-institutionalised US population aged 2 months+.  Response rate 64% [[8]](#footnote-9)∇ | | | 78 GB Men | | | | 3214 Non GB Men | | | 2 | | | **Suicide attempt [lifetime]**  **Suicidal ideation [lifetime]**  **DSM-III-R Major Depression [lifetime]**  DSM-III-R Any Mood Disorder⊥[[9]](#footnote-10) [lifetime] |
| Cochran, Sullivan, Mays (2003) [15]  USA  and  Mays & Cochran (2001) [16]  USA | Community sample of men and women aged 25-74 years. | | | Random digit dialled sample. Response rate 61% | | | 37 LB Women  37 GB Men | | | | 1604 Non LB Women  1239 Non GB  Men | | | 3 | | | **DSM-III-R Major Depression [<12 months]**  **DSM-III-R Generalised Anxiety Disorder**  **[<12 months]**  Any Psychiatric Disorder[[10]](#footnote-11) [<12 months]  **DSM-IV Alcohol Dependence [<12 months]**  **DSM-IV Drug Dependence [<12 months]** |
| Faulkner AH, Cranston K (1998) [17]  USA | Male and female students aged 14-17 years. | | | Representative, population-based sample of high school students drawn from 1993 Risk Behaviour Survey Participation rate 55 % | | | 56 Lesbians/Gay Men  49 Bisexual Men/ Women | | | | 1563  Non LGB Men/Women | | | 3 | | | **Suicide attempt [<12 months]**  **Suicidal ideation [<12 months]** |
| Fergusson, Horwood, Beautrais (1999) [18]  New Zealand | Birth cohort, men and women aged 21 years. | | | 21 year follow up of Christchurch Health and Development Study birth cohort. Study includes data from 80% follow up of original cohort. | | | 17 LB Women  11 GB Men | | | | 979 Non LGB Men/Women | | | 2, 3 | | | **Suicide attempt [lifetime]**  **Suicidal ideation [lifetime]**  **DSM-III-R Major Depression [lifetime]**  **DSM-III-R Generalised Anxiety Disorder [lifetime]**  **DSM-III-R Substance Use Disorder [lifetime]** |
| Study Description  Author/Year/  Country | | Nature of population | | | Sampling/recruitment technique  and response rate | | | Numbers of participants | | | | | Definition of sexual orientation | | Study outcomes of interest | | |
| Gilman, Cochran, Mays, Hughes, Ostrow, Kessler (2001) [19]  USA | | Community sample of men and women aged 15 to 54 years. | | | National random sample, representative of US population.  Response rate 82% | | | 51 LB Women  74 GB Men | | 2475 Non LB Women  2310 Non GB  Men | | | 2 | | **Suicide attempt [<12 months]**  **Suicidal ideation [<12 months]**  **DSM-III-R Major Depression [<12 months]**  **DSM-III-R Any Anxiety Disorder**  **[<12 months]**  DSM-III-R Any Mood Disorder [<12 months]  **DSM-III-R Substance Use Disorder [<12 months]**  **DSM-III-R Alcohol Dependence [<12 months]**  **DSM-III-R Drug Dependence [<12 months]** | | |
| Gruskin, Hart, Gordon, Ackerson  (2001) [20]  USA | | Community sample of women aged 20 - 65+ years. | | | Random sample of 34000 members of Kaiser Permanente stratified by age and sex. Response rate 48% | | | 120 LB Women | | 7793 Non LB Women | | | 3 | | **Alcohol Misuse[[11]](#footnote-12) [<12 months]** | | |
| Herrell, Goldberg, True, Ramakrishnan, Lyons, Eisen,  Tsuang (1999) [21] USA | | Male-male twin pairs aged 40-60 years | | | Analytic sample of 103 male-male twins born between 1939 and 1957 in which one member of the pair reported male sex partners after age 18 years drawn from twin registry. Response rate 72%[[12]](#footnote-13) | | | 103 Gay male twin pairs | | 103 Non Gay male twin pairs | | | 2 | | **Suicide attempt [lifetime]**  **Suicidal ideation [lifetime]** | | |
| Jorm, Korten, Rodgers, Jacomb, Christensen (2002) [22]  Australia | | Electoral roll sample of men and women aged 20-24 and 40-44 in two states in Australia. | | | Random sample from electoral roll that answered sexual orientation question.  Response rate 59% in 20-24 and 65% in 40-44.[[13]](#footnote-14)∇ | | | 78 Lesbians/  Gay Men  71 Bisexual Men/Women | | 4824 Non LGB Men/Women | | | 3 | | **Suicidal ideation**  **[<12 months]**  Goldberg Anxiety and Depression Scales  **Alcohol use disorder (AUDIT[[14]](#footnote-15))** | | |
| King, McKeown Warner, Ramsay, Johnson, Cort, Wright, Blizard, Davidson (2003) [1] UK | | Community sample of men and women aged 16+ years. | | | Six snowball waves of lesbian gay and heterosexuals recruited through advertising in national press, in venues and through LGB organisations. Response rate N/A | | | 430 Lesbians  656 Gay men | | 588 Non Lesbian Women  505 Non Gay Men | | | 3, 4 | | **Deliberate self harm [lifetime]**  Common Mental Disorder Clinical Interview Schedule]  **Alcohol use disorder (AUDIT)** | | |
| Study Description  Author/Year/  Country | | | Nature of population | | | Sampling/recruitment technique  and response rate | | | Numbers of participants | | | | | Definition of sexual orientation | | Study outcomes of interest | |
| Mathy (2002a) [23]  North America | | | Men and women selected from Human Sexuality Study 2000  No age range given | | | Two web sites: 1) every 1000^th^ visitor N = 7544 2) every visitor from other N = 33,391  Response rate 25% | | | 1048 LB Women  3754 GB Men | | | 5499 Non LB Women  25622 Non GB Men | | 3 | | **Suicide attempt [lifetime]**  **Suicidal ideation [lifetime]** | |
| Matthews, Hughes, Johnson, Razzano, Cassidy (2002) [24]  USA | | | Lesbians and their female work-role counterpart aged less than 30 to 60+ years. | | | Multi-site survey using snowball sampling in 1994-1996. Response rate 48%[[15]](#footnote-16)∇ | | | 550 Lesbians | | | 279 Non L Women | | 1, 2 | | **Suicide attempt [lifetime]**  **Suicidal ideation [lifetime]** | |
| McCabe, Boyd, Hughes, D'Arcy (2003) [25]  USA | | | Male and female students aged 17+ years | | | Random sample assigned to mail or internet survey mode. Response rate 52% | | | 65 LB Women  54 GB Men | | | 2042 Non LB Women  1446 Non GB Men | | 3 | | **Alcohol Misuse**  **[<12 months]** | |
| McCabe, Hughes, Boyd (2004) [26]  USA | | | Female students aged 17+ years | | | Random sample assigned to mail or internet survey mode. Response rate 59% | | | 49 Bisexual Women | | | 2042 Non B Women | | 3 | | **Suicidal ideation [<12 months]**  Alcohol misuse (CAGE[[16]](#footnote-17)) | |
| Nawyn, Richman, Rospenda, Hughes (2000) [27] USA | | | Employees at American University.  Age not provided | | | Sampled using unspecified method.  Response rate 52% | | | 40 LB Women  56 GB Men | | | 1254 Non LB Women  1075 Non GB Men | | 3 | | Alcohol misuse (MAST[[17]](#footnote-18)) | |
| Remafedi, French, Story, Resnick, Blum (1998) [28]  USA | | | Male and female students aged 12 to 17 years from one US state. | | | Modified, stratified cluster sampling.  Response rate over 95%[[18]](#footnote-19)∇ | | | 38 Lesbians  81 Gay men  144 Bisexual Women  131 Bisexual Men | | | 152 Non LB Women  184 Non GB Men | | 3, 4 | | **Suicide attempt [<12 months]**  **Suicidal ideation [<12 months]** | |
| Robin, Brener, Donahue, Hack, Hale, Goodenow (2002) [29]  USA | | | Male and female students aged 14-17 years. | | | Representative, population-based sample of high school students drawn from the 1995 and 1997 Risk Behaviour Survey  Response rate 63-72 % ^∇^ | | | 355 Lesbians/ Gay Men  458 Bisexual Men/ Women | | | 10821  Non LGB Men/Women | | 2,3 | | **Suicide attempt [<12 months]** | |
| Russell & Joyner (2001) [30]  USA | | | Male and female students aged 12 to 17 years. | | | More than 12000 adolescents, from 134 schools, stratified by grade and sex, were selected  Response rate cannot be calculated ^∇^ | | | 414 LB Women  453 GB Men | | | 5840 Non LB Women  5233 Non GB Men | | 1, 2 | | **Suicide attempt [<12 months and lifetime]**  **Suicidal ideation [<12 months and lifetime]** | |
| Study Description  Author/Year/  Country | | | Nature of population | | | Sampling/recruitment technique  and response rate | | | Numbers of participants | | | | | Definition of sexual orientation | | Study outcomes of interest | |
| Sandfort, de Graaf, Bijl, Schnabel (2001) [31]  Netherlands  and  Sandfort, de Graaf, Bijl (2003) [32]  Netherlands | | | Community sample of men and women aged 18-64 years. | | | Multi-stage, stratified, random sample of households in the Netherlands. One respondent was randomly selected from each household. To optimise response and to compensate for possible seasonal influences, the initial fieldwork was between February and December 1996.  Response rate 70%[[19]](#footnote-20)∇ | | | 43 Lesbians  82 Gay Men | | | 3077 Non Lesbian Women  2796 Non Gay Men | | 2 | | **DSM-III-R Major Depression [<12 months and lifetime]**  **DSM-III-R Generalised Anxiety Disorder**  **[<12 months and lifetime]**  DSM-III-R Any Mood Disorder  [<12 months and lifetime]  **DSM-III-R Substance Use Disorder**  **[<12 months and lifetime]**  **DSM-III-R Alcohol Dependence**  **[<12 months and lifetime]**  **DSM-III-R Drug Dependence**  **[<12 months and lifetime]** | |
| Skegg, Nada-Raja, Dickson, Paul, Williams (2003) [33]  New Zealand | | | Longitudinal birth cohort of men and women aged 26 years. | | | Sample comprised survivors at study time point born 1972 to 1973.  Response rate 93%[[20]](#footnote-21)∇ | | | 9 Lesbians  110 Bisexual Women  8 Gay men  45 Bisexual men | | | 343 Non LB Women  427 Non GB men | | 1 | | **ICD-9 Deliberate self harm [lifetime]**  **Suicide attempt [lifetime]**  **Suicidal ideation [<12 months]** | |
| Wichstrom & Hegna (2003) [34]  Norway | | | Prospective cohort of male and female students aged 12 -20 years. | | | Sample from 67 representative high schools at follow-up 3 (1999).  Response rate 68%. | | | 190 Lesbians/Gay Men/Bisexual Men and Women | | | 2924 Non LGB Men/Women | | 1, 2 ,4 | | Suicide attempt [lifetime] | |
| Drabble et al. (2005) [35] USA | | | Population based national alcohol survey | | | National sample in the USA using random digit dialling | | | 324 homosexual identified, bisexual identified, and heterosexual identified with same sex partners (the latter were combined with bisexual in our analysis) | | | 6924 exclusively heterosexual men and women | | 2, 3 | | **DSM-IV Alcohol Dependence**  **[12 months]** | |

1. 1) Same sex attraction; 2) Same sex behaviour; 3) Self identification as LGB; 4) Kinsey scale. *Note more than one definition may be used* [↑](#footnote-ref-2)
2. We only report outcomes that meet our definition and were extracted for analysis [↑](#footnote-ref-3)
3. Denominator stated as 688 in article [↑](#footnote-ref-4)
4. **Bold** indicates inclusion in meta analysis [↑](#footnote-ref-5)
5. Center for Epidemiological Studies Depression Scale [↑](#footnote-ref-6)
6. ^+^ Short Form-36

   ^∇^ Denominator is those who were able to be categorised on the definition of their sexuality [↑](#footnote-ref-7)
7. ^^ [↑](#footnote-ref-8)
8. ^^ Denominator is those who were able to be categorised on the definition of their sexuality [↑](#footnote-ref-9)
9. ^⊥?^ Insufficient studies for a meta analysis of Any Mood Disorder [↑](#footnote-ref-10)
10. A composite score comprising Major Depression, GAD, Panic Disorder, Alcohol Dependence and Drug Dependence [↑](#footnote-ref-11)
11. Misuse based on recommended alcohol limits >14 drinks per week for women and >21 drinks per week for men [↑](#footnote-ref-12)
12. Only an approximate rate provided [↑](#footnote-ref-13)
13. ^^ Denominator is those who were able to be categorised on the definition of their sexuality [↑](#footnote-ref-14)
14. Alcohol Use Disorders Identification Test [↑](#footnote-ref-15)
15. ^^ Denominator is those who were able to be categorised on the definition of their sexuality [↑](#footnote-ref-16)
16. ‘CAGE’ Alcohol Screening Test [↑](#footnote-ref-17)
17. Michigan Alcohol Screening Test [↑](#footnote-ref-18)
18. ^^ [↑](#footnote-ref-19)
19. ^^ Denominator is those who were able to be categorised on the definition of their sexuality [↑](#footnote-ref-20)
20. ^^ [↑](#footnote-ref-21)
